# Understanding Antibiotic Use in Companion Animals: A Literature Review Identifying Avenues for Future Efforts

**DOI:** 10.3389/fvets.2021.719547

**Published:** 2021-10-08

**Authors:** Alice C. Tompson, Ana L. P. Mateus, Dave C. Brodbelt, Clare I. R. Chandler

**Affiliations:** ^1^London School of Hygiene and Tropical Medicine, London, United Kingdom; ^2^VEEPH Group, Department of Pathobiology and Population Sciences, Royal Veterinary College, London, United Kingdom

**Keywords:** antibiotic, antimicrobial consumption, companion animal, epidemiology, qualitative research

## Abstract

Addressing antibiotic use is essential to tackle antimicrobial resistance, a major human and animal health challenge. This review seeks to inform stewardship efforts in companion animals by collating research insights regarding antibiotic use in this group and identifying overlooked avenues for future research and stewardship efforts. The development of population-based methods has established that antibiotics are frequently used in companion animal care. Research insights are also contributing toward an in-depth comprehension of the contexts to antibiotic use. Qualitative approaches, for example, have enabled a nuanced understanding in four key areas: interactions with owners, clinical and financial risk management, time pressures, and clinic dynamics. This review identifies that much of the existing research frames antibiotic use as the result of choices made by the individuals at the interface of their use. Future research and policy endeavours could look beyond the moment of prescribing to consider the societal structures and networks in which companion animal antibiotic use is entangled. A diversification in research approaches and frameworks through which antibiotic use is understood will facilitate the identification of additional targets for stewardship initiatives beyond providing information and awareness campaigns.

## Introduction

Antibiotic use in both human and animal populations is coming under increasing scrutiny due to its role as a driver in the emergence of antimicrobial resistance (AMR), a phenomena through which bacteria have or acquire the ability to withstand the effects of these important medicines (1). As well as sharing lives and living spaces, people and companion animals also share resistant bacteria (2, 3) and medicines, with many antibiotic classes being used in both populations (4, 5). Use of antibiotics in companion animals could be an important driver of AMR relevant to human health, that has largely been overlooked by major policy responses (3, 6). As a consequence, addressing antibiotic use–particularly those deemed to be of highest priority critically importance (HPCIAs)–in companion animals is becoming of increasing policy interest (7–9).

This review provides an overview of the evolving approaches for understanding of antibiotic use in companion animals and the insights arising from this research. This is with two aims: firstly, to provide a companion animal specific synthesis, as whilst a recent systematic review synthesised quantitative and qualitative findings regarding the non-clinical factors influencing veterinarian antibiotic use (10), a companion animal sub-analysis was not undertaken. An in-depth overview of this unique context–less driven by the “rational” cost-benefit decisions of the livestock sector, for example–in which antibiotic use is situated remains needed. Secondly, this review considers the vantage points used to understand antibiotic use. It is concerned with not only *what* these studies found in terms of explaining antibiotic use, but also *how* they framed this phenomenon and went about investigating it. This is important because it, in turn, shapes the proposed responses to antibiotic use. It therefore aids the identification of currently overlooked vantage points and avenues for future interventions.

## Study Design

This review is a narrative review conducted in a systematic manner. Studies were identified via searches of PubMed, CAB Abstracts, Google scholar, reference list and citation searching. The International Standard Randomised Controlled Trials Number clinical trial registry was scrutinised and investigators of trials in progress contacted for further details. Searches were last conducted in April 2021 and comprised of search terms including veterinarian OR veterinary) AND (companion OR small OR pet^*^) AND (antibiotic^*^ OR antimicrobial^*^).

Supplementary Table 1 describes the eligibility criteria applied. Eligible studies must consider antibiotic use by companion animal veterinarians. Commentaries or reviews with no primary data were excluded, as were papers describing resistant bacteria found in companion animals; the latter were deemed beyond the scope of this article. No arbitrary start date for studies to be considered for inclusion was set. Data regarding the study authors, population, methods, intervention design and findings were extracted into a standardised template in excel and synthesised. Data extraction was not checked by a second reviewer, nor were formal quality and risk of bias assessments undertaken.

In order to consider how studies have framed antibiotic use in companion animals, a new framework based on social research into antibiotic use around the world, was applied (11). The framework proposes three complimentary vantage points from which to understand and address antibiotic use (Table 1).

**Table 1:** Vantage points from which to understand antibiotic use in companion animals. Adapted from Tompson and Chandler (11).

**Vantage point**	**Description**
Practices	•Focuses on antibiotic use at end user level and the context in which this occurs. •Interventions often change behaviour and practice by altering the context in which individuals make decisions about antibiotic use. •Interventions might include: improving communication between veterinarians and owners, providing information on medicines, awareness/ education tailored to local understandings of ill health and treatment, adjusting practitioner renumeration arrangements.
Structures	•Considers antibiotic use as a product of the economic and political conditions of modern societies. •Interventions modify these underlying conditions and so reduce the need for antibiotics to be used as quick fixes for productivity, hygiene, and inequality (12). •Interventions might include: improving providing affordable, accessible veterinary care through social insurance, addressing intensive breeding practices, and social demands for unhealthy companion animal breeds.
Networks	•Draws attention to the networks of materials and ideas that connect humans and non-humans-animals, medicines, microbes, technologies-that extend through time and space far beyond the moment of antibiotic use. •Interventions redesign networks and tracks that define antibiotic use. •Interventions might include: reconfiguring veterinary pathways/protocols, rerouting the supply chain of veterinary products and medicines; adjusting accountability frameworks.

## Findings

The characteristics of the 83 studies identified in the preparation of this review are summarised in the Supplementary Table 2. There has been an increase in the number of papers published in this field, particularly since 2017. Research has been primarily conducted in Europe and Australia. Few studies took place in Africa and South America, with none identified in Asia. In recent years, there has been a move toward qualitative studies and those considering the perspectives and experiences of social actors other than veterinarians.

### Establishing Antibiotic Usage Patterns

Over the last 20 years or so, our epidemiological understanding of antibiotic use in companion animals has greatly advanced. Early efforts relied on sales records to produce population level estimates of usage as part of integrated antibiotic use surveillance programmes in Nordic countries (13–15). Such approaches are able to monitor longitudinal changes in use (16, 17) and make international comparisons (18) but are unable to comment on the clinical context of their usage or its appropriateness. Meanwhile, smaller scale studies described patterns of antibiotic use based on data extracted from the clinical or prescribing records of veterinary hospitals and clinics (19–28). These methods produce local insights into antibiotic practices but the use of teaching hospitals, in some case, limits the generalisability of their findings. A third methodological approach was to survey veterinarians regarding their typical antibiotic usage (29–36) or about their usage in recent patient cases (37–40). These enabled the estimation of usage levels and exploration of variation in practices but are subject to selection bias as volunteering veterinarians might not be representative of their profession and, furthermore, their responses might be influenced by social desirability bias.

Supported by developments in information technology infrastructures, two UK-based surveillance systems have been crucial in furthering our understanding of antibiotic use in companion animals. VetCompass^TM^ (Royal Veterinary College, RVC) and SAVSNET (Liverpool University) collate data from the electronic patient records of hundreds of first opinion veterinary clinics (41, 42) enabling larger, multi-site studies of antibiotic use (4, 43–45) and quantification of variation in use between clinics (44–46), a phenomenon also observed in other countries (47–49). Analyses have also considered the utilisation of specific antibiotics (46, 50, 51) and their role in specific conditions (52–56). In Australia, the VetCompass^TM^ methodology has recently been extended to include the automation of free text labelling to describe diagnostic categories (57–59), paving the way for studies investigating appropriate use in large datasets derived from clinical records (49).

In the UK, VetCompass^TM^ and SAVSNET studies agree that antibiotics are frequently used in companion animal care with broad-spectrum amoxicillin-clavulanate being the most commonly prescribed agent in dogs. Over a two-year period, one in four UK dogs (25.2%, 95% confidence interval (CI): 25.1–25.3%) received antibiotics (4). HPCIAs have been estimated to make up around 5–6% of total events with fluoroquinolones being the most commonly used HPCIA in dogs, constituting ~4–5% of total antibiotic events (4, 45). One in five cats (20.6%, 95% CI: 20.5–20.7) received antibiotics over a 2-year time frame with HPCIA use accounting for 34.6% of antibiotic events (4). This was largely driven by the use of cefovecin, an injectable, long acting formulation that was the most frequently used antibiotic in cats (4, 45). In both UK dog and cat populations, antibiotic use is gradually declining (45, 60), a trend that has also been observed in other countries (17, 18, 61, 62). Although, there has been a reduction in antibiotic use, there is still room for further improvement.

### Explaining Antibiotic Use

The following sections are organised by the methodological approach of the reviewed research.

#### Quantitative Methods

To date, surveys of veterinarians form the majority of the literature and have been used to investigate the contexts in which antibiotics are used. They have acknowledged that this extends beyond solely clinical factors with respondents being asked to report or rank factors influencing their antibiotic use (33, 34, 63–68). These efforts all fall within the practices perspective of understanding antibiotic utilisation (11).

Supplementary Tables 3–5 provide summaries of the studies that ranked factors influencing antibiotic use or produced estimates of association with non-clinical factors. A survey of UK companion animal veterinarians found that clinical presentation was the most important factor, followed by bacterial culture, ease of antibiotic administration, and financial constraints, with client expectations being the least important (69). Echoing these findings, client expectations for antibiotics have been ranked as a minor influence across a number of studies and settings. These include UK veterinarians in perioperative situations (63), those working at US veterinary teaching hospitals (34, 64), a variety of settings in Australia (68, 70) and the Netherlands (71), and first opinion clinics in Belgium (33). Whilst a high proportion of a sample of Australian veterinarians reported experiencing client pressure to prescribe, they also stated that their clients' and colleagues' expectations had minimal influence on their antibiotic use (70). However, in another Australian study, the most frequently selected factor limiting antibiotic stewardship was client pressure (24% of 97 respondents) with client finances in third place (11%) (72). Veterinarians also rated economic factors as of low importance when deciding whether/which antibiotics to use in European settings (33, 71). When surveyed, only a small minority (9%) of a sample of Flemish veterinarians (*n* = 284) felt financial restrictions—presumably of the client—were an important factor (33). However, in a survey of veterinarians (*n* = 54) in South Africa, a middle-income country, 77% reported that cost influenced their choice of antibiotics (73). In terms of profit from antibiotic sales, almost three-quarters of veterinarians surveyed in Australia (72%, *n* = 172) strongly disagreed that this influenced their decision to prescribe (72).

#### Veterinarian Characteristics

Information about the veterinarian respondents themselves has also been collected in order to ask questions such as, “What type of veterinarian is more likely to use antibiotics appropriately?” In a UK study, Hughes et al. (69) reported that the odds of clinicians (*n* = 460) working in a veterinary referral hospital prescribing the incorrect antibiotics dose were half of those of veterinarians who did not [odds ratio (OR): 0.5, 95% CI: 0.3–0.8], whilst locums were more likely to prescribe antibiotics off-label than clinic partners (OR: 4.8, 95% CI: 1.3–18.0). In Australia, Hardefeldt et al. (32) found that in response to hypothetical clinical vignettes, 88% of the reported use of HPCIAs was contained within the replies of 50% of surveyed companion animal veterinarians (with the other half of respondents reporting just 12% of HPCIA use) (total sample size = 892). However, no differences between the year of graduation or postcode-derived socio-economic variables were observed between these groups. Across all veterinary sectors, a systematic review concluded that socio-demographic characteristics did not appear to influence antibiotic use (10).

#### Use of Information

When investigating which veterinarians are more likely to use antibiotics appropriately, another area of interest has been the information sources they draw upon, e.g., clinical experience, pre-/post-qualification education, and the published literature (33, 63, 69, 73–76). UK companion animal veterinarians reporting use of pharmaceutical company information were found to be more likely to prescribe second- and third-generation cephalosporins compared to those who did not (OR: 1.87, 95% CI: 1.04–3.37) (69). However, when asked directly, Australian veterinarians stated that manufacturer promotional material had minimal or no impact on their antibiotic prescribing (70), a finding echoed by UK experts (77). These findings highlight a limitation of relying on self-reported data and expecting veterinarians to be aware of–and able to articulate–the prevailing conditions shaping their antibiotic practices.

Linked to the interest in the role of information and education in guiding appropriate antibiotic use, a number of surveys have studied veterinarians' knowledge, attitudes, and beliefs surrounding antibiotic use and AMR (68, 70, 78, 79). When Australian veterinarians of all sectors were surveyed, Norris et al. (70) found that the greatest disconnect between personal use of antibiotics and concerns about AMR was shown by companion animal veterinarians. Recently, the adequacy of veterinary undergraduate education in this regard has come under scrutiny, with student knowledge regarding appropriate antibiotic use being deployed as a surrogate measure for subsequent practice (80, 81). These surveys are typically characterised by low response rates introducing the possibility of self-selection bias. Therefore, it is questionable how representative and generalisable the results are.

#### The Role of Clinic Policies and Guidelines

Another form of information available to veterinarians are clinical guidelines and policies. The introduction of guidelines is positioned as a key step in optimising antibiotic use (82). Professional organisations such as the British Veterinary Association, the British Small Animal Veterinary Association, and the Federation of European Companion Veterinary Associations have provided guidance on appropriate antibiotic use (83–85). Surveys–now possibly outdated–suggest that a minority of UK small animal clinics have local antibiotic use policies (65, 69), an observation replicated elsewhere in the world (36, 66, 72, 76, 86, 87). Encouragingly, two-thirds of a sample (*n* = 71) of UK veterinary students had heard of the British Veterinary Association's “Responsible Use of Antimicrobials” guideline (80). However, a survey of 254 American veterinarians conducted in 2015 found 88% were unaware of the existence of professional antibiotic use guidelines, with over three-quarters welcoming more guidance in this area (78). In Australia, livestock veterinarians typically indicated guideline recommendations as having a “strong” influence on their antibiotic decisions, whilst their companion animal counterparts rated them as a “moderate” influence (70). This suggests that the impact of introducing guidelines might vary between veterinary sectors.

Jessen et al. (66) investigated the impact of the introduction of Danish prescribing guidelines for companion animal veterinarians (*n* = 151). Almost two-thirds (65%) of the respondents reported the guidelines had altered their habits. The main barriers to adherence were: confidence in old prescribing practices (46%); unavailability of licenced products (34%); difficulties dosing the drug (e.g., due to limited tablet sizes) (31%); costs (30%); lack of time for consulting the guidelines (25%); a limited number of antibiotics available on site (23%); and owners' difficulties in administering drugs (18%). These findings hint at the potential clash between standardised, expert opinion-based guidelines and individual veterinarian's empirical experience amassed over their career working as a largely autonomous professional (8).

#### The Interaction of Multiple Contextual Factors

One might ponder how well the complex on-the-ground realities of providing companion animal veterinary care are represented by the ranking of individual, stand-alone factors. Two recent quantitative analyses have used more complex approaches to investigate how factors might combine to produce antibiotic use.

Hopman et al. (71) investigated the links between veterinarian demographics, attitudes, working environment, and antibiotic use through a Categorical Principal Component Analysis of survey data. The result was a model with three dimensions: The first— “social responsibility” —was characterised by well-considered antibiotic prescribing, self-confidence, independence, and recognition of their role in public and animal health, whilst being uninfluenced by owners' demands and working in a well-equipped clinic. This dimension was positively associated with more experienced veterinarians and working in dedicated companion animal clinics or referral centres. The second dimension— “scepticism” —was illustrated through the attitude of “no harm done by trying antibiotics.” It was linked to risk avoidance behaviours at an individual animal level and ignorance of the possible AMR risks derived from antibiotic use in companion animals. This dimension was positively associated with being male and a more experienced veterinarian. The final dimension-fear of the possible consequences of not prescribing antibiotics—illustrated by a “better safe than sorry” habit especially around surgery and was associated with veterinarians working full-time and in rural clinics (71).

Extending their work describing levels of antibiotic use, the SAVSNET team used multivariable mixed effects logistic regression to investigate dog-, clinic-, and owner-related factors influencing the likelihood of prescribing antibiotics in over a quarter of a million consultations with unwell dogs attending 379 clinics (51). They found that dogs who were vaccinated (OR: 0.93, 95% CI: 0.90–0.95), insured (OR: 0.87, 95% CI: 0.84–0.90), and neutered (OR: 0.90, 95% CI: 0.88–0.92) were less likely to receive systemic antibiotics than those who were not. This suggests a link between owners engaging with preventative healthcare measures and not using antibiotics, although this cross-sectional study is unable to demonstrate a causal pathway. In terms of clinic-related factors, those treating companion animals and large animals were associated with significantly increased odds of systemic antibiotic use compared with companion animal-only practices (OR: 1.15, 95% CI:1.01–1.30). Clinics accredited by the Royal College of Veterinary Surgeons (RCVS) were also less likely to prescribe a systemic antibiotic (OR: 0.79, 95% CI: 0.68–0.92). No clear association between antibiotic use and the owner-related factors considered—their neighbourhood deprivation, companion animal population density, and rural or urban status—were observed. However, the authors noted that the simplified measure of deprivation used (a collapsed version of the Index of Multiple Deprivation) may struggle to describe the realities of owners' circumstances (51).

These studies begin to consider how contextual factors combine to produce the environment in which antibiotics are used. It is debatable how well quantitative methods can describe these complex, shifting, socially situated practices and in the last few years, there has been an increased use of qualitative methods to study this phenomenon.

### Qualitative Studies

Over ten years after the initial quantitative investigations into antibiotic use in companion animals, Mateus et al. (88) published the first qualitative study, in which UK veterinarians were interviewed. This was followed by similar projects in Australia (72), the Netherlands (89), and the United States (90). Recently, researchers have considered the perspectives of other social actors by interviewing companion animal owners in the UK (91) and the US (92), enabling a more rounded understanding of decisions to use antibiotics. There has also been a slight shift away from research being conducted by veterinarians situated in veterinary schools (93). For example, a multidisciplinary team—including social scientists—in Scotland have undertaken a programme of research into antibiotic use in companion animals. Their interest has extended beyond veterinarians (94) to veterinarians and owners (95) and owners at home (90). They have also considered the perspectives of policy makers and the “experts” (77). In doing so, antibiotic “misuse” is rendered less of a clinical problem that veterinarians, alone, are able to define, study, and propose answers to.

**Figure 1:**
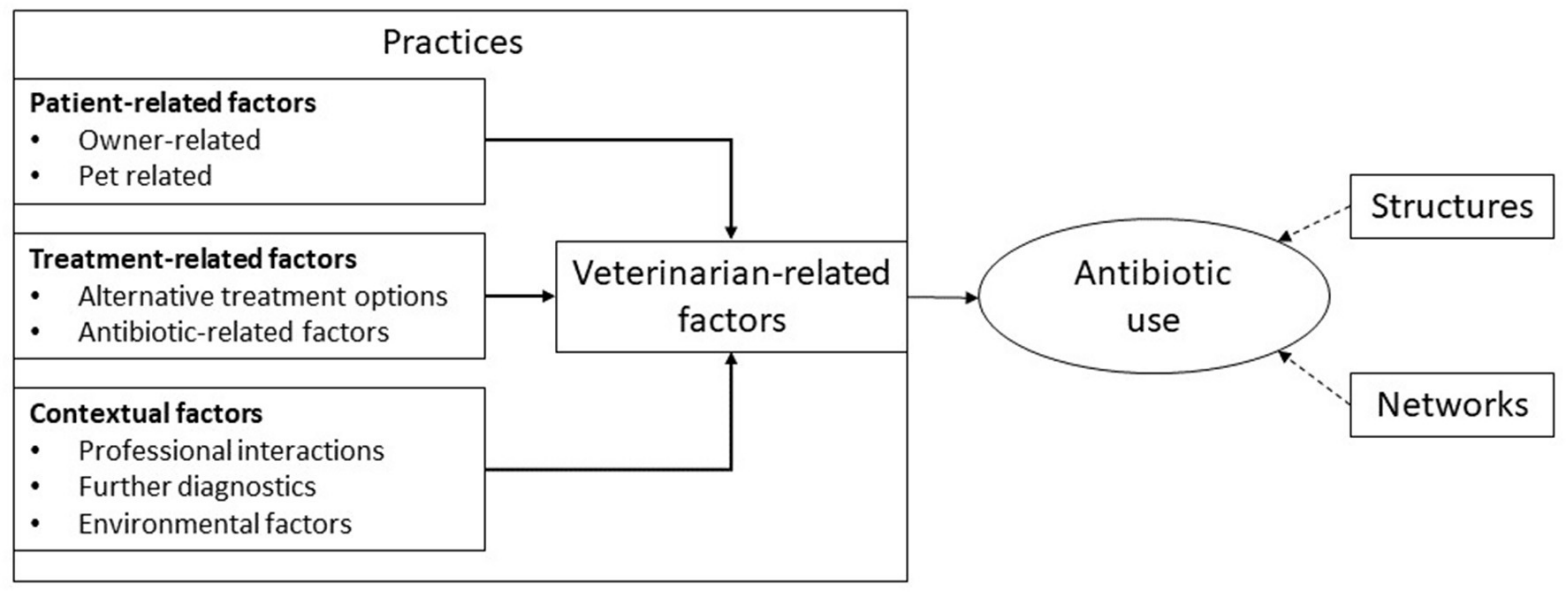
A typical framework through which companion animal antibiotic use has been explained. Adapted from Hopman et al. (89).

These qualitative studies use descriptive, thematic coding to produce lists of factors or themes shaping antibiotic use with authors making limited use of social theory (Table 2). They typically adopt a practices perspective—in which antibiotic use is positioned as the result of choices made by individuals (11) (Figure 1). In the section below, the contribution these qualitative studies have made in providing a more nuanced understanding of antibiotic use based on insight provided in the following areas: interactions with owners, clinical and financial risk management, time pressures, and clinic dynamics.

**Table 2:** A summary of qualitative and mixed methods studies investigating antibiotic use in companion animals.

**References**	**Theoretical approach**	**Key themes**
**Veterinarians**		
Mateus et al. (88) United Kingdom	Thematic analysis to identify factors associated with antimicrobial usage.	Intrinsic factors (linked to the veterinarian). Extrinsic factors I (antimicrobial characteristics, workplace and colleagues at work). Extrinsic factors II (characteristics of pet owners and animals).
Cartelet et al. (93) United Kingdom	Thematic analysis of veterinarians' experience prescribing antimicrobials, attitudes about antimicrobials and AMR	Uncertainty, risk and clinical decision making. Professionalism-authority vs. stewardship? The client as both adversary and collaborator in consultations. Stewardship and stress: a difficult balance to strike.
King et al. (94) United Kingdom	Thematic analysis to identify behavioural drivers of veterinary prescribing (barriers and facilitators).	Clinical need for antibiotics. Responding to clients. Confirming diagnosis. Determining dose, duration and type of antibiotic. Preventing infection around surgery (with attendant antibiotic prescribing).
Hopman et al. (89) Netherlands	An iterative analysis guided by the questions “which factors influence the decision to prescribe antimicrobials” and “which factors influence which antimicrobial to prescribe.”	Veterinarian-related factors. Patient-related (i.e., owner- and animal-related) factors. Treatment-related factors (i.e., alternative treatment options and antimicrobial-related factors). Contextual factors (i.e., professional interactions, further diagnostics and environmental factors).
Hardefeldt et al. (72) Australia	Thematic analysis to identify barriers to and enablers of implementing antimicrobial stewardship programs in veterinary practices.	Encounters with and perceptions of AMR. Client expectations of antimicrobial treatment and competition between clinics. Costs associated with diagnostic testing. Lack of resources and access to training. Hierarchical structure of many clinics.
Lavigne et al. (90) United States	Thematic analysis to identify the influence of financial considerations on antibiotic use.	Client willingness to pay for diagnostic testing or treatment interferes with the ability of veterinarians to make appropriate antibiotic use decisions. Antibiotic selection is limited by fear of expiration and the resulting financial losses. Restricting antibiotic use to “appropriate use” carries financial risks.
**Veterinarians and owners**		
Smith et al. (95) United Kingdom	A behavioural framework to identify key behaviours emerging from participant accounts which were amenable to change.	Perceived pressures from owners. Opportunities to discuss AMR and appropriate prescribing with owners. Owner's expectations. Owner's understanding of AMR.
**Owners**		
Dickson et al. (91) United Kingdom	An interpretative phenomenological analysis of the relationship between owners and their companion animals as a key context for AMR-related behaviours.	“They're my fur babies”: unconditional love and anthropomorphism. “They share everything with you”: affection and transmission behaviours. “We would err on the side of caution”: decision making and antibiotic use'.
Redding et al. (92) United States	“Conventional content analysis” of knowledge of and attitudes towards the judicious use of antimicrobials.	Owner knowledge of antimicrobials and AMR. Following directions when administering antimicrobials to companion animals. Expectations for antimicrobials. Perceptions of initiatives for the judicious use of antimicrobials. Use of antimicrobials when their efficacy is ambiguous.
**Policy makers, academics and leaders**		
Currie et al. (77) United Kingdom	Delphi study to identify veterinary behaviours which experts believe contribute to AMR and form vital aspects of antimicrobial stewardship.	The behaviours perceived to be most influential were antibiotic prescribing (poor choice and unnecessary use) and interactions with clients. Other veterinary behaviours perceived as less important were interactions with veterinary colleagues; infection control practices; and diagnostic tests to confirm infection.

#### Interactions With Owners

Much of this research has been undertaken from the perspective of veterinarians. Based on interviews with companion animal veterinarians, Mateus et al. (88) identified three main ways in which owners shape antibiotic prescribing: their (veterinarian-perceived) compliance with drug administration instructions; their willingness or ability to pay for medicines or diagnostics; and their expectations and the pressure they exert on veterinarians to provide antibiotics. These findings were replicated by Hopman et al. (89) whose veterinarian interviewees also described the influence of owner convenience. Lavigne et al. (90) reiterated how owner reluctance to pay for diagnostic testing and treatment act as barriers to “appropriate” antibiotic use. They reported how economic concerns may prompt animal euthanasia if antibiotic use is restricted (90).

Qualitative studies add more nuance to the representations of veterinarians being under a constant, unyielding pressure to prescribe antibiotics from owners (96). When interviewed, UK veterinarians reported that it was “increasingly rare” for owners to directly ask for antibiotics (94) and, if they did, most would accept veterinarian recommendation that they might not be needed (93). A study that considered the perspectives of both veterinarians and owners found that the former felt the latter applied pressure for antibiotics, whilst the latter felt the former were responsible for “overuse” (95). Furthermore, the perceived owner anxiety and expectation for antibiotics were often inferred by veterinarians, rather than explicitly stated by owners (95).

Clients—and their desire for their companion animal to recover quickly—have been framed as an obstacle to appropriate antibiotic prescribing (95). Having interviewed veterinarians, King et al. (94) described how owners see antibiotics as a clear pathway to their animals' recovery, avoiding having to “wait it out” to see if they recovered. Mateus et al. (88) reported a mismatch between what veterinarians felt they should be doing as professionals with regards to antibiotic use and their perception of what owners expected, i.e., affordable care and a “quick fix” for their companion animal.

Studies of owner perspectives have provided further insight into this “pressure.” Smith et al. (95) described how owners were pushed into making financial and other sacrifices to ensure their companion animal—a family member—got better. The owners interviewed described experiencing their animal's suffering viscerally (95). Dickson et al. (91) reported how owners anticipated feelings of “intolerable guilt” if their companion animal died due to their complacency. Minimising their companion animal's suffering and getting their veterinarian to realise how sick their pet was were key concerns amongst US owners (92). Adopting the “better safe than sorry” approach may help reduce the immediate anxiety of owners whilst supporting antibiotic use (91).

#### Managing Clinical and Financial Risk

Echoing this “better safe than sorry” approach of owners, veterinarian interviewees also described cautionary prescribing of antibiotics to mitigate against potential future clinical complications, especially if existing patterns of antibiotic use were known to work (94). Cartelet et al. (93) reported how veterinarian's decision making is fraught with uncertainty and the focus is typically on lowering the perceived risk to the individual companion animal. A Dutch veterinarian described how “I think it is because it has become a habit and because one is afraid to leave it out in case it would then go wrong” (88. p. 109). In addition to managing clinical risk, antibiotics were also used to help reduce the risk of dissatisfied owners seeking care elsewhere (88). Smith et al. (95) described the tension between appropriate antibiotic use, client satisfaction, and running a viable business. In the United States, Lavigne et al. explained the potential financial penalties associated by veterinarians with not meeting owner demands for antibiotics (90). They also reported how concerns regarding financial losses arising from product expiration influenced the types and amounts of antibiotics stocked and therefore available for use by veterinarians (90).

#### Time Pressures

Qualitative studies have provided insight into the time pressures faced by companion animal veterinarians. Time constraints—linked to fixed duration consultations—hamper in-depth conversations and the undertaking of in house diagnostic testing to guide treatment plans (88). Veterinarians also described the implicit assumption that owners would want the most effective and quickest treatment in order to return their companion animal to good health (94). Dutch veterinarians reported prescribing antibiotics as a “quick fix” for themselves and/or owners (89).

#### Clinic Dynamics

Beyond the consultation, qualitative studies have begun to investigate the broader context in which antibiotics are used. Hardefeldt et al. (72) reported the hierarchical structure of many clinics to be a major barrier to antimicrobial stewardship, although no further details were provided. Mateus et al. (88) described the general influence of senior veterinarians have in mentoring and supporting less experienced colleagues in handling complex clinical cases, whilst Hopman et al. (89) found younger graduates were more likely to be prudent users of antibiotics. King quotes a senior veterinarian who explained “the new grads are initially more prone to not give antibiotics because they were taught, well-actually it's bad, and they stand their ground more. But then as they get in to practice and get more experience and maybe they just get worn down or maybe the daily life. then they start giving antibiotics more loosely” [King et al., (94), p. 5]. Meanwhile Hopman et al. (89) quotes a more junior colleague who said “look, I am always happy to talk about the matter [antibiotic use], but it remains his word. Nevertheless, to put things bluntly, I must do what he says if I want to keep my job” (88. p. 110).

These qualitative studies have provided additional insight into antibiotic use by companion animal veterinarians and have begun to consider broader social context. However, echoing the quantitative studies, their framing is typically orientated around practices and have made limited used of social theory as a “tool” to help unpick the complexities of antibiotic use.

## Discussion

This review has drawn together the growing body of literature seeking to understand antibiotic use in companion animals. Research has used quantitative and, increasingly, qualitative methods to investigate the contexts and social practises surrounding their utilisation, recognising that it is not a purely clinical matter. Despite the increasing range of methods used, research efforts remain orientated around understanding antibiotic use through the lens of practices—concerned with the individuals involved in decision-making about their use (11).

### From Reported Behaviours to Enacted Practices

In addition to the shared framing of antibiotic use, the studies identified adopt the same philosophical starting point of positivism: as with much public health research, they expect the social world to be understandable by revealing sets of factors or rules, in the same way that the natural world can be understood. It presumes that these rules can become known through self-reporting; that individuals, when asked, can account for their behaviour. However, this mode of understanding the social world has been countered, partly as knowledge and beliefs rarely predict behaviour, but also because social phenomena tend to operate in registers that are invisible and illegible to those operating within them (97–99).

For example, this review collated mixed findings regarding the role of owners in influencing veterinarian antibiotic use. Some studies concluded this influence is unimportant whilst others positioned owners as a major barrier to enacting antibiotic stewardship. Qualitative studies have begun to render visible circulating expectations in this regard but interviewees may struggle to fully articulate the complexities of veterinarian-owner-animal encounters and the taken-for-granted, “common sense” at play. Ethnography, including participant observation, has recently been used to investigate antibiotic use in companion animals for the first time (46, 100). Such methodologies are able to study enacted practices—rather than reported behaviours—rendering visible the easily overlooked and thus making a valuable contribution in understanding antibiotic use and identifying targets for intervention (101).

### Overlooking Social Networks and Structures

Reflecting the prevailing practices vantage point (11) identified by this review, the recent systematic review of veterinarian antibiotic use structured its findings as factors intrinsic or extrinsic to the veterinarian (10). Whilst the latter included structural factors such as biosecurity and hygiene, economic incentives and the role of the pharmaceutical industry, these are positioned as shaping the conscious and sub-conscious context in which veterinarians make decisions regarding their treatment plans. However, social scientists have proposed that not all antibiotic use flows through individuals and further consideration needs to be given to networks: the channels that mean antibiotics are present or absent, or their use is expected or unexpected, in certain times and places (Table 1) (11).

A growing number of researchers working in the livestock sector have considered the networks of people, farm animals, microbes, living conditions, markets, value chains, supply chains and regulations through which antibiotic use patterns emerge thus demonstrating the value of decentring the individual (102–105). Future research in the companion animal sector could adopt similar following methodologies to consider the flow of antibiotics and ideas of their “appropriate use” through space and time, far beyond the moment of their prescription in the veterinary clinic (12).

Describing a social phenomenon requires analysis that moves beyond individual accounts to consider collectively produced understandings of illness, health and medicines use (106). Situating accounts with other materials and observations can help render visible and legible the social, political, and economic structures that shape antibiotic use (107, 108). For example, the social demands for specific dog breeds have resulted in the intensification and commodification of certain forms of canine bodies (109, 110). This in turn shapes the form of care—including antibiotic use—that companion animal veterinarians are required to practise (46). The prevailing practices vantage point has caused these wider political and economic imperatives in society that foster antibiotic use in companion animals to be overlooked and future research projects should address this knowledge gap.

### Providing Information to Address Antibiotic Use

Despite a deepening contextual understanding of antibiotic use in companion animals—including insights into the roles of interactions with owners, risk management, time pressures, and clinic dynamics—existing research efforts to intervene have centred on education and information provision to alter the decision making of veterinarians (17, 20, 67, 111, 112) or owners (113). Whilst providing access to guidelines to veterinarians appears to cause some reduction in antibiotic use, it seems insufficient to fully optimise practices (111, 112). However, drawing firm conclusions from these studies is hampered by their design—before and after protocols make it impossible to exclude the effects of other longitudinal changes (20) whilst short-term follow-up limits our understanding of the sustainability of such interventions (67). Further high-quality evaluations are needed (114).

Two larger scale studies have evaluated the impact of multi-faceted interventions centred around information provision in first opinion clinics in the Netherlands (*n* = 44) (115) and the UK (*n* = 60) (116). Hopman et al. (115) evaluated a stewardship programme—including benchmarking activities, veterinarian education, owner information sheets and social pledges—via a stepped wedged design, whereby clusters of clinics were randomised to receive the intervention at regular intervals following baseline assessment. A 15% reduction in total clinic antibiotic use was modelled (95% CI: 7–22%, *p* < 0.01). However, it is unclear which part(s) of the intervention were responsible for the changes seen and if such an intensive intervention—including the provision of locum staff to cover clinical duties—is feasible or affordable in “real world” settings.

As part of their randomised controlled trial, Singleton et al. (116) used the SAVSNET system to identify clinics with higher than average HPCIA use. Clinics randomised to the two intervention arms were provided with educational materials, and those in the intensive intervention arm were additionally enrolled in an education and reflection programme including in-depth benchmarking information, and meetings with clinical managers seeking to identify and address factors contributing to their antibiotic use. At these sites, there was a 23.5% reduction in canine consultations in which HPCIAs were prescribed (0.5% of total consultations, 95% CI: 0.4–0.6, *p* = 0.004) and a 39.0% reduction in the equivalent feline consultations (4.4%, 95% CI: 3.4–5.3, *p* < 0.001). A smaller reduction in HPCIA use was recorded in the less intensive intervention arm (*p* = 0.700 in dogs; *p* = 0.030 in cats). This trial also monitored the potential unintended consequences of intervening: no statistically significant changes in euthanasia rates were observed to coincide with a reduction in HPCIA use. This is an important insight to reassure frontline veterinarians and owners about changing ways of using antibiotics, especially given their role in risk management identified by qualitative studies. Further description and evaluation of the contents of the meetings could aid a better understanding of the means by which this intervention had impact: perhaps by via addressing the barriers identified in these meetings and/or having regular meetings with a senior manager who was scrutinising antibiotic use in the clinic? It would be valuable to explore whether a peer-led project would have the same impact and the sustainability of such interventions.

These studies suggest developing policies and guidelines, and providing training is an important step in optimising antibiotic use. However, a lack of knowledge regarding their “appropriate” use or awareness of AMR is not the sole driver of usage patterns (11). The central role given to information provision and education in efforts to address antibiotic use in companion animals to date could reflect the use of specific research tools, namely surveys of knowledge, awareness and practice, used to characterise and explain the phenomenon (117). Future studies in this field might reflect upon how research questions influence proposed solutions, and consider a further diversification of approaches. The interest in providing information has partly obscured other contextual issues raised in the qualitative studies, such as time pressures or the use of antibiotics “just in case.” As acknowledged in these studies, these issues cannot be fully resolved solely by educating veterinarians and/or owners about appropriate use. Future interventions could, for example, evaluate the impact of altering the risk-benefit ratio veterinarians encounter when deciding whether to supply antibiotics and assess the impact of stewardship initiatives on client turnover and the financial sustainability of the clinic.

The literature included in this review largely focusses on Europe and Australia and therefore it is questionable whether its findings can be generalised to the other geographical and economic contexts, particularly given that access to veterinary care and the social standing of companion animals differ around the world. Future research conducted in a diverse range of settings would help develop our understanding of antibiotic usage levels in companion animal populations and the local contexts surrounding their use. Developing an in-depth understanding, perhaps through longitudinal studies following animals over their life span, might help to elucidate potential alternatives to antibiotic use such as engaging owners in preventive veterinary care (51).

Understanding and addressing antibiotic use in human healthcare has been the focus of considerable efforts (118) that could inform initiatives in the companion animal veterinary sector. Such an approach would align with One Health responses to AMR that call for co-operation between human and animal health domains (119). However, the companion animal sector is assembled in a markedly different way: veterinary care is provided to a partially insured population largely through a network of private clinics. In some countries, such as the UK, antibiotic sales form an important income stream for veterinary clinics, a factor that has not required consideration by the healthcare system there (the National Health Service or NHS). Drawing on insights from human healthcare in countries such as the United States with private systems serving partly insured populations may help to supplement the existing research literature in companion animals.

## Conclusion

The research literature investigating antibiotic use in companion animals has diversified beyond quantitative methods in recent years, enabling a more nuanced understanding of this social phenomenon. However, research efforts have remained orientated around a positivist philosophy and the practices lens (11). Further social science informed endeavours would be a welcome addition to the growing array of quantitative and, increasingly, qualitative studies listing factors held to shape antibiotic use. Considering antibiotic use through the lenses of networks and structures could be a valuable addition to efforts to understand antibiotic use in this setting and contribute toward the identification of stewardship approaches beyond providing information to optimise antibiotic use.

## Author Contributions

AT wrote the first draft. CC, DB, and AM commented on the draft. All authors approve the submitted version of the manuscript and developed the remit of the review and contributed toward identifying relevant literature.

## Funding

This research was financially supported by a Bloomsbury Colleges Ph.D. studentship and an Antibiotics Research UK research grant (ANTSRG 05/2018).

## Conflict of Interest

The authors declare that the research was conducted in the absence of any commercial or financial relationships that could be construed as a potential conflict of interest.

## Publisher's Note

All claims expressed in this article are solely those of the authors and do not necessarily represent those of their affiliated organizations, or those of the publisher, the editors and the reviewers. Any product that may be evaluated in this article, or claim that may be made by its manufacturer, is not guaranteed or endorsed by the publisher.
