# Supplementary material for: Understanding Antibiotic Use in Companion Animals: A Literature Review Identifying Avenues for Future Efforts
Source: Front Vet Sci. 2021 Oct 8;8:719547. doi: 10.3389/fvets.2021.719547 (PMC8531418; doi:10.3389/fvets.2021.719547)
Supplement: Supplementary file 1 [file Data_Sheet_1.docx]

Supplementary Material

# Supplementary table 1. Eligibility criteria for the review

| **Population** | - Must include companion animals - Both inpatient and outpatient populations were considered. - Studies considering only large animals, such as horses or livestock, were excluded |
| --- | --- |
| **Intervention** | - Seeks to quantify antibiotic use - Seeks to explain antibiotic use - Seek to alter antibiotic use |
| **Study designs** | - Observational studies - (Randomised controlled) trials - Before and after comparisons (e.g. field intervention studies) - Qualitative studies - Mixed methods studies - Delphi consensus techniques - Ethnographic studies |

**Supplementary table 2.** Summary of the characteristics of the identified research studies. # = dogs; ~ =cats; #~= dogs & cats; * = companion animals. Annual surveillance reports are not included.

| Year of publication | Lead author | Country/region | | | | | | | | Settings | | | | | | Approach | | | Methods | | | | | | Sample size | | | | | | | | |
| --- | --- | --- | --- | --- | --- | --- | --- | --- | --- | --- | --- | --- | --- | --- | --- | --- | --- | --- | --- | --- | --- | --- | --- | --- | --- | --- | --- | --- | --- | --- | --- | --- | --- |
|  |  | UK | Scandinavia | Rest of Europe | North America | Australia/New Zealand | Asia | Africa | South America | Pharmacies/wholesalers | University/referral hospitals | First opinion clinics and hospitals | Academia/government experts | University | Non-veterinary-owners | Quantitative | Mixed Methods | Qualitative | Manual case review, observation | Survey | Database study | Intervention, pre/post comparison | Interviews, focus groups | Genotyping | <50 | <100 | <500 | <1,000 | <5,000 | <10,000 | <50,000 | <100,000 | 100,000+ |
| 2001 | Odensvik^#~^ (1) |  |  |  |  |  |  |  |  |  |  |  |  |  |  |  |  |  |  |  |  |  |  |  | Not reported | | | | | | | | |
|  | Watson^#^ (2) |  |  |  |  |  |  |  |  |  |  |  |  |  |  |  |  |  |  |  |  |  |  |  |  |  |  |  |  |  |  |  |  |
| 2004 | Rantala^#^ (3) |  |  |  |  |  |  |  |  |  |  |  |  |  |  |  |  |  |  |  |  |  |  |  |  |  |  |  |  |  |  |  |  |
| 2005 | Heuer^#~^ (4) |  |  |  |  |  |  |  |  |  |  |  |  |  |  |  |  |  |  |  |  |  |  |  | Not reported | | | | | | | | |
|  | Holso^#~^ (5) |  |  |  |  |  |  |  |  |  |  |  |  |  |  |  |  |  |  |  |  |  |  |  |  |  |  |  |  |  |  |  |  |
| 2006 | Hill^*^ (6) |  |  |  |  |  |  |  |  |  |  |  |  |  |  |  |  |  |  |  |  |  |  |  |  |  |  |  |  |  |  |  |  |
|  | Weese^#~^ (7) |  |  |  |  |  |  |  |  |  |  |  |  |  |  |  |  |  |  |  |  |  |  |  |  |  |  |  |  |  |  |  |  |
| 2009 | Black^#^ (8) |  |  |  |  |  |  |  |  |  |  |  |  |  |  |  |  |  |  |  |  |  |  |  |  |  |  |  |  |  |  |  |  |
|  | Regula^#~^ (9) |  |  |  |  |  |  |  |  |  |  |  |  |  |  |  |  |  |  |  |  |  |  |  |  |  |  |  |  |  |  |  |  |
|  | Thomson^~^ (10) |  |  |  |  |  |  |  |  |  |  |  |  |  |  |  |  |  |  |  |  |  |  |  |  |  |  |  |  |  |  |  |  |
| 2010 | German^#^ (11) |  |  |  |  |  |  |  |  |  |  |  |  |  |  |  |  |  |  |  |  |  |  |  |  |  |  |  |  |  |  |  |  |
| 2011 | Escher^#~^ (12) |  |  |  |  |  |  |  |  |  |  |  |  |  |  |  |  |  |  |  |  |  |  |  |  |  |  |  |  |  |  |  |  |
|  | Mateus^#~^ (13) |  |  |  |  |  |  |  |  |  |  |  |  |  |  |  |  |  |  |  |  |  |  |  |  |  |  |  |  |  |  |  |  |
|  | Radford^*^ (14) |  |  |  |  |  |  |  |  |  |  |  |  |  |  |  |  |  |  |  |  |  |  |  |  |  |  |  |  |  |  |  |  |
|  | Wayne^#^ (15) |  |  |  |  |  |  |  |  |  |  |  |  |  |  |  |  |  |  |  |  |  |  |  |  |  |  |  |  |  |  |  |  |
| 2012 | Baker^#^ (16) |  |  |  |  |  |  |  |  |  |  |  |  |  |  |  |  |  |  |  |  |  |  |  |  |  |  |  |  |  |  |  |  |
|  | Hughes^#~^ (17) |  |  |  |  |  |  |  |  |  |  |  |  |  |  |  |  |  |  |  |  |  |  |  |  |  |  |  |  |  |  |  |  |
|  | Knights^#~^ (18) |  |  |  |  |  |  |  |  |  |  |  |  |  |  |  |  |  |  |  |  |  |  |  |  |  |  |  |  |  |  |  |  |
|  | Murphy^#~^ (19) |  |  |  |  |  |  |  |  |  |  |  |  |  |  |  |  |  |  |  |  |  |  |  |  |  |  |  |  |  |  |  |  |
|  | Pleydell^*^ (20) |  |  |  |  |  |  |  |  |  |  |  |  |  |  |  |  |  |  |  |  |  |  |  |  |  |  |  |  |  |  |  |  |
| 2013 | De Briyne^*^ (21) |  |  |  |  |  |  |  |  |  |  |  |  |  |  |  |  |  |  |  |  |  |  |  |  |  |  |  |  |  |  |  |  |
|  | Kvaale^#^ (22) |  |  |  |  |  |  |  |  |  |  |  |  |  |  |  |  |  |  |  |  |  |  |  | Not reported | | | | | | | | |
| 2014 | De Briyne^*^ (23) |  |  |  |  |  |  |  |  |  |  |  |  |  |  |  |  |  |  |  |  |  |  |  |  |  |  |  |  |  |  |  |  |
|  | Mateus^#~^ (24) |  |  |  |  |  |  |  |  |  |  |  |  |  |  |  |  |  |  |  |  |  |  |  |  |  |  |  |  |  |  |  |  |
|  | Summers^#^ (25) |  |  |  |  |  |  |  |  |  |  |  |  |  |  |  |  |  |  |  |  |  |  |  |  |  |  |  |  |  |  |  |  |
| 2015 | AVMA^#~^ (26) |  |  |  |  |  |  |  |  |  |  |  |  |  |  |  |  |  |  |  |  |  |  |  |  |  |  |  |  |  |  |  |  |
|  | Jacob^#~^ (27) |  |  |  |  |  |  |  |  |  |  |  |  |  |  |  |  |  |  |  |  |  |  |  |  |  |  |  |  |  |  |  |  |

**Supplementary table 2 (cont.).** Summary of the characteristics of the identified research studies. # = dogs; ~ =cats; #~= dogs & cats; * = companion animals. Annual surveillance reports are not included.

| Year of publication | Lead author | Country/region | | | | | | | | Settings | | | | | | Approach | | | Methods | | | | | | Sample size | | | | | | | | |
| --- | --- | --- | --- | --- | --- | --- | --- | --- | --- | --- | --- | --- | --- | --- | --- | --- | --- | --- | --- | --- | --- | --- | --- | --- | --- | --- | --- | --- | --- | --- | --- | --- | --- |
|  |  | UK | Scandinavia | Rest of Europe | North America | Australia/New Zealand | Asia | Africa | South America | Pharmacies/wholesalers | University/referral hospitals | First opinion clinic and hospitals | Academia/government experts | University | Non-veterinary-owners | Quantitative | Mixed Methods | Qualitative | Manual case review | Survey | Database study | Intervention, pre/post comparison | Interviews, focus groups, observation | Genotyping | <50 | <100 | <500 | <1,000 | <5,000 | <10,000 | <50,000 | <100,000 | 100,000+ |
| 2016 | Buckland^*^ (28) |  |  |  |  |  |  |  |  |  |  |  |  |  |  |  |  |  |  |  |  |  |  |  |  |  |  |  |  |  |  |  |  |
|  | Fowler^*^ (29) |  |  |  |  |  |  |  |  |  |  |  |  |  |  |  |  |  |  |  |  |  |  |  |  |  |  |  |  |  |  |  |  |
|  | Lloyd^*^ (30) |  |  |  |  |  |  |  |  |  |  |  |  |  |  |  |  |  |  |  |  |  |  |  |  |  |  |  |  |  |  |  |  |
| 2017 | Barbarossa^*^ (31) |  |  |  |  |  |  |  |  |  |  |  |  |  |  |  |  |  |  |  |  |  |  |  |  |  |  |  |  |  |  |  |  |
|  | Barzelai^#^ (32) |  |  |  |  |  |  |  |  |  |  |  |  |  |  |  |  |  |  |  |  |  |  |  |  |  |  |  |  |  |  |  |  |
|  | Burke^~^ (33) |  |  |  |  |  |  |  |  |  |  |  |  |  |  |  |  |  |  |  |  |  |  |  |  |  |  |  |  |  |  |  |  |
|  | Chipangura^#^ (34) |  |  |  |  |  |  |  |  |  |  |  |  |  |  |  |  |  |  |  |  |  |  |  |  |  |  |  |  |  |  |  |  |
|  | Hardefeldt^#~^ (35) |  |  |  |  |  |  |  |  |  |  |  |  |  |  |  |  |  |  |  |  |  |  |  |  |  |  |  |  |  |  |  |  |
|  | Hardefeldt^#~^ (36) |  |  |  |  |  |  |  |  |  |  |  |  |  |  |  |  |  |  |  |  |  |  |  |  |  |  |  |  |  |  |  |  |
|  | Jessen^*^ (37) |  |  |  |  |  |  |  |  |  |  |  |  |  |  |  |  |  |  |  |  |  |  |  |  |  |  |  |  |  |  |  |  |
|  | Sarrazin^#~^ (38) |  |  |  |  |  |  |  |  |  |  |  |  |  |  |  |  |  |  |  |  |  |  |  |  |  |  |  |  |  |  |  |  |
|  | Singleton^#~^ (39) |  |  |  |  |  |  |  |  |  |  |  |  |  |  |  |  |  |  |  |  |  |  |  |  |  |  |  |  |  |  |  |  |
| 2018 | Cartelet^*^ (40) |  |  |  |  |  |  |  |  |  |  |  |  |  |  |  |  |  |  |  |  |  |  |  |  |  |  |  |  |  |  |  |  |
|  | Currie^*^ (41) |  |  |  |  |  |  |  |  |  |  |  |  |  |  |  |  |  |  |  |  |  |  |  |  |  |  |  |  |  |  |  |  |
|  | Dyar^*^ (42) |  |  |  |  |  |  |  |  |  |  |  |  |  |  |  |  |  |  |  |  |  |  |  |  |  |  |  |  |  |  |  |  |
|  | Gomez^#^ (43) |  |  |  |  |  |  |  |  |  |  |  |  |  |  |  |  |  |  |  |  |  |  |  |  |  |  |  |  |  |  |  |  |
|  | Hardefeldt^*^ (44) |  |  |  |  |  |  |  |  |  |  |  |  |  |  |  |  |  |  |  |  |  |  |  |  |  |  |  |  |  |  |  |  |
|  | Hardefeldt^*^ (45) |  |  |  |  |  |  |  |  |  |  |  |  |  |  |  |  |  |  |  |  |  |  |  |  |  |  |  |  |  |  |  |  |
|  | Hardefeldt^#~^ (46) |  |  |  |  |  |  |  |  |  |  |  |  |  |  |  |  |  |  |  |  |  |  |  |  |  |  |  |  |  |  |  |  |
|  | Hopman^*^ (47) |  |  |  |  |  |  |  |  |  |  |  |  |  |  |  |  |  |  |  |  |  |  |  |  |  |  |  |  |  |  |  |  |
|  | King^*^ (48) |  |  |  |  |  |  |  |  |  |  |  |  |  |  |  |  |  |  |  |  |  |  |  |  |  |  |  |  |  |  |  |  |
|  | Smith^*^ (49) |  |  |  |  |  |  |  |  |  |  |  |  |  |  |  |  |  |  |  |  |  |  |  |  |  |  |  |  |  |  |  |  |
|  | Sorensen^#^ (50) |  |  |  |  |  |  |  |  |  |  |  |  |  |  |  |  |  |  |  |  |  |  |  |  |  |  |  |  |  |  |  |  |
|  | Van Cleven^#~^ (51) |  |  |  |  |  |  |  |  |  |  |  |  |  |  |  |  |  |  |  |  |  |  |  |  |  |  |  |  |  |  |  |  |
|  | Zhuo^*^ (52) |  |  |  |  |  |  |  |  |  |  |  |  |  |  |  |  |  |  |  |  |  |  |  |  |  |  |  |  |  |  |  |  |

**Supplementary table 2 (cont.).** Summary of the characteristics of the identified research studies. # = dogs; ~ =cats; #~= dogs & cats; * = companion animals. Annual surveillance reports are not included.

| Year of publication | Lead author | Country/region | | | | | | | | Settings | | | | | | Approach | | | Methods | | | | | | Sample size | | | | | | | | |
| --- | --- | --- | --- | --- | --- | --- | --- | --- | --- | --- | --- | --- | --- | --- | --- | --- | --- | --- | --- | --- | --- | --- | --- | --- | --- | --- | --- | --- | --- | --- | --- | --- | --- |
|  |  | UK | Scandinavia | Rest of Europe | North America | Australia/New Zealand | Asia | Africa | South America | Pharmacies/wholesalers | University/referral hospitals | First opinion clinic and hospitals | Academia/government experts | University | Non-veterinary-owners | Quantitative | Mixed Methods | Qualitative | Manual case review, | Survey | Database study | Intervention, pre/post comparison | Interviews, focus groups, observations | Genotyping | <50 | <100 | <500 | <1,000 | <5,000 | <10,000 | <50,000 | <100,000 | 100,000+ |
| 2019 | Dickson^*^ (53) |  |  |  |  |  |  |  |  |  |  |  |  |  |  |  |  |  |  |  |  |  |  |  |  |  |  |  |  |  |  |  |  |
|  | Ekakoro^*^ (54) |  |  |  |  |  |  |  |  |  |  |  |  |  |  |  |  |  |  |  |  |  |  |  |  |  |  |  |  |  |  |  |  |
|  | Hopman^*^ (55) |  |  |  |  |  |  |  |  |  |  |  |  |  |  |  |  |  |  |  |  |  |  |  |  |  |  |  |  |  |  |  |  |
|  | Hopman^*^ (56) |  |  |  |  |  |  |  |  |  |  |  |  |  |  |  |  |  |  |  |  |  |  |  | Not reported | | | | | | | | |
|  | Hopman^*^ (57) |  |  |  |  |  |  |  |  |  |  |  |  |  |  |  |  |  |  |  |  |  |  |  |  |  |  |  |  |  |  |  |  |
|  | Hopman^*^ (58) |  |  |  |  |  |  |  |  |  |  |  |  |  |  |  |  |  |  |  |  |  |  |  | Not reported | | | | | | | | |
|  | Norris^*^ (59) |  |  |  |  |  |  |  |  |  |  |  |  |  |  |  |  |  |  |  |  |  |  |  |  |  |  |  |  |  |  |  |  |
|  | Redding^*^ (60) |  |  |  |  |  |  |  |  |  |  |  |  |  |  |  |  |  |  |  |  |  |  |  |  |  |  |  |  |  |  |  |  |
|  | Redding^*^ (61) |  |  |  |  |  |  |  |  |  |  |  |  |  |  |  |  |  |  |  |  |  |  |  |  |  |  |  |  |  |  |  |  |
|  | Schmitt^~^ (62) |  |  |  |  |  |  |  |  |  |  |  |  |  |  |  |  |  |  |  |  |  |  |  |  |  |  |  |  |  |  |  |  |
|  | Singleton^*^ (63) |  |  |  |  |  |  |  |  |  |  |  |  |  |  |  |  |  |  |  |  |  |  |  |  |  |  |  |  |  |  |  |  |
|  | Singleton^*^ (64) |  |  |  |  |  |  |  |  |  |  |  |  |  |  |  |  |  |  |  |  |  |  |  |  |  |  |  |  |  |  |  |  |
|  | Singleton^#^ (65) |  |  |  |  |  |  |  |  |  |  |  |  |  |  |  |  |  |  |  |  |  |  |  |  |  |  |  |  |  |  |  |  |
|  | Singleton^*^ (66) |  |  |  |  |  |  |  |  |  |  |  |  |  |  |  |  |  |  |  |  |  |  |  |  |  |  |  |  |  |  |  |  |
| 2020 | Hardefeldt^~^ (67) |  |  |  |  |  |  |  |  |  |  |  |  |  |  |  |  |  |  |  |  |  |  |  |  |  |  |  |  |  |  |  |  |
|  | Hubbuch^~^ (68) |  |  |  |  |  |  |  |  |  |  |  |  |  |  |  |  |  |  |  |  |  |  |  |  |  |  |  |  |  |  |  |  |
|  | Hur^#~^ (69) |  |  |  |  |  |  |  |  |  |  |  |  |  |  |  |  |  |  |  |  |  |  |  |  |  |  |  |  |  |  |  |  |
|  | Joosten^#~^ (70) |  |  |  |  |  |  |  |  |  |  |  |  |  |  |  |  |  |  |  |  |  |  |  |  |  |  |  |  |  |  |  |  |
|  | Lehner^#^ (71) |  |  |  |  |  |  |  |  |  |  |  |  |  |  |  |  |  |  |  |  |  |  |  |  |  |  |  |  |  |  |  |  |
|  | Lutz^#^ (72) |  |  |  |  |  |  |  |  |  |  |  |  |  |  |  |  |  |  |  |  |  |  |  |  |  |  |  |  |  |  |  |  |
|  | Robbins^#~^ (73) |  |  |  |  |  |  |  |  |  |  |  |  |  |  |  |  |  |  |  |  |  |  |  |  |  |  |  |  |  |  |  |  |
|  | Singleton^#~^ (74) |  |  |  |  |  |  |  |  |  |  |  |  |  |  |  |  |  |  |  |  |  |  |  |  |  |  |  |  |  |  |  |  |
|  | Stallwood^~^ (75) |  |  |  |  |  |  |  |  |  |  |  |  |  |  |  |  |  |  |  |  |  |  |  |  |  |  |  |  |  |  |  |  |
|  | Tompson^#^ (76) |  |  |  |  |  |  |  |  |  |  |  |  |  |  |  |  |  |  |  |  |  |  |  |  |  |  |  |  |  |  |  |  |
|  | Valiakos^*^ (77) |  |  |  |  |  |  |  |  |  |  |  |  |  |  |  |  |  |  |  |  |  |  |  |  |  |  |  |  |  |  |  |  |
| 2021 | Alcantara^*^ (78) |  |  |  |  |  |  |  |  |  |  |  |  |  |  |  |  |  |  |  |  |  |  |  |  |  |  |  |  |  |  |  |  |
|  | Chirollo^#~^ (79) |  |  |  |  |  |  |  |  |  |  |  |  |  |  |  |  |  |  |  |  |  |  |  |  |  |  |  |  |  |  |  |  |
|  | Galarce^*^ (80) |  |  |  |  |  |  |  |  |  |  |  |  |  |  |  |  |  |  |  |  |  |  |  |  |  |  |  |  |  |  |  |  |
|  | Lavigne* (81) |  |  |  |  |  |  |  |  |  |  |  |  |  |  |  |  |  |  |  |  |  |  |  |  |  |  |  |  |  |  |  |  |
|  | Samuels^*^ (82) |  |  |  |  |  |  |  |  |  |  |  |  |  |  |  |  |  |  |  |  |  |  |  |  |  |  |  |  |  |  |  |  |
|  | Singleton^#~^ (83) |  |  |  |  |  |  |  |  |  |  |  |  |  |  |  |  |  |  |  |  |  |  |  |  |  |  |  |  |  |  |  |  |

**Supplementary table 3a.** An overview of quantitative studies estimating the association of factors with features of antibiotic use (n=11)

| **Author, year** | **Country** | **Population** | **Outcome Measure** | **Methods** | **Sample size** |
| --- | --- | --- | --- | --- | --- |
| Hughes,  2012 (17) | UK | Companion animal vets working in first opinion and hospitals identified by professional register | Use of unlicensed antimicrobials  Use of 2^nd^ & 3^rd^ gen cephalosporins  Use of fluoroquinolones  Only reports statistically significant results. | Survey, multivariable logistic regression models | 444 veterinarians |
| Hardefeldt, 2017 (35) | Australia | Companion animal vets working in first opinion and hospitals identified by professional register | Surgical guidelines compliance | survey, multivariable logistic regression models | 886 veterinarians |
| Hardefeldt, 2017 (36) | Australia | Companion animal vets working in first opinion and hospitals identified by professional register | High user of antimicrobials | Survey | 892 veterinarians |
| Hardefeldt, 2018 (44) | Australia | Veterinary students | Guideline compliance | Survey | 476 veterinary students |
| Hardefeldt, 2018 (46) | Australia | Insured cats and dogs | Total antimicrobial usage, CIA usage | Logistic regression model using insurance claim data | 813,172 dog-years and 129,232 cat-years |
| Van Cleven, 2018 (51) | Belgium | First opinion companion animal veterinarians | Guideline compliance, use of first-choice agents | Survey, univariable analysis | 223 veterinarians |
| Ekakoro, 2019 (54) | US | Veterinarians working at a single teaching hospital | Concern about AMR | survey, multivariable logistic regression models | 121 veterinarians |
| Hopman, 2019 (58) | Netherlands | First opinion companion animal veterinary clinics | Total antimicrobial use, 3^rd^ choice antimicrobial use | Multivariable regression analysis using prescribing data | 44 clinics |
| Hur,  2020 (69) | Australia | Companion animal veterinary consultations | Antimicrobial prescribing, HPCIA prescribing | Prescribing data | 4,400,519 consultations |
| Lutz,  2020 (72) | Switzerland | First opinion companion animal veterinary clinics and veterinary hospitals | Guideline compliance for selected canine diseases | Prescribing data | 1,065 cases |
| Singleton, 2020 (74) | UK | First opinion companion animal veterinary consultations | Systemic antimicrobial use, HPCIA use | Multivariable mixed effects logistic regression of prescribing data | 392,682 consultations |

AMR: Antimicrobial resistance; CIA: Critically Important Antimicrobials; HPCIA: Highest Priority Critically Important Antimicrobials.

**Supplementary table 3b.** An overview of the association between investigated factors and features of higher antibiotic use – owner and veterinarian related factors (n=8)

| **Author, year** | **Owner** | **Veterinarian** | | | | | | |
| --- | --- | --- | --- | --- | --- | --- | --- | --- |
|  | **Insurance status** | **Gender** | **Age** | **Experience** | **Role** | **Country of graduation** | **Information sources used** | **Post-graduate qualification** |
| Hughes,  2012 (17) |  |  |  | More recent graduates↓ | Locum 🡩 |  | Pharmaceutical company info 🡩 |  |
| Hardefeldt, 2017 (35) |  | Not SS |  | Recent graduates (post 2011) 🡩 | Not SS |  |  | Not SS |
| Hardefeldt 2017 (36) |  |  |  | Not SS |  |  |  |  |
| Hardefeldt, 2018 (44) |  |  |  | Not SS |  |  |  |  |
| Van Cleven, 2018 (51) |  |  |  |  |  |  | Scientific literature 🡫 |  |
| Ekakoro,  2019 (54) |  |  |  | More recent graduates 🡫 |  |  | CPD 🡫 |  |
| Hopman,  2019 (58) |  | Not SS |  |  |  | Not SS |  |  |
| Singleton,  2020 (74) | Insured 🡫 (for systemic antibiotics)  Insured 🡩  (for HPCIAs) |  |  |  |  |  |  |  |

Not SS: Not statistically significant; 🡩Associated with a statistically significant increase of use; 🡫Associated with a statistically significant reduction of use

CPD: Continuing Professional Development; HPCIA: Highest Priority Critically Important Antimicrobials.

**Supplementary table 3b.** An overview of the association between investigated factors and features of higher antibiotic use – clinic related factors (n=9) Information about the role of specific geographic regions was not included due to limited relevence to a general audience.

| **Author, year** | **Clinic factors** | | | | | | | | |
| --- | --- | --- | --- | --- | --- | --- | --- | --- | --- |
|  | **Clinic type** | **Case mix** | **RCVS accreditation status** | **Clinic size** | **Location** | **Antibiotic use policy** | **Offers alternative medicines** | **Serves kennels** | **Serves breeders** |
| Hughes,  2012 (17) | Referral hospital 🡫 |  | Accredited 🡩 |  |  |  |  |  |  |
| Hardefeldt, 2017 (35) | Not SS | Mixed 🡩 |  | <3 vets 🡩 | Rural/urban: Not SS | Not SS |  |  |  |
| Hardefeldt, 2017 (36) |  | Not SS |  |  | Deprivation: Not SS |  |  |  |  |
| Hardefeldt, 2018 (44) |  | Not SS |  |  |  |  |  |  |  |
| Hardefeldt, 2018 (46) |  |  |  |  | Metropolitan 🡩 |  |  |  |  |
| Hopman,  2019 (58) |  |  |  | Not SS | Not SS |  | Not SS | Not SS | Not SS |
| Hur,  2020 (69) | Emergency and referral centres 🡩 |  |  |  | Urban 🡩 |  |  |  |  |
| Lutz,  2020 (72) | Referral hospital 🡫 |  |  |  |  |  |  |  |  |
| Singleton, 2020 (74) |  | Mixed 🡩 | Accredited 🡫 |  |  |  |  |  |  |

Not SS: Not statistically significant; 🡩 associated with a statistically significant increase of use; 🡫 associated with a statistically significant reduction of use

RCVS: Royal College of Veterinary Surgeons.

**Supplementary table 4.** Studies ranking factors influencing antibiotic use (n=7)

| **Study details** | Whether or not to use antibiotics | Antibiotic choice in perioperative situations | Choice of antibiotics in general | | | | |
| --- | --- | --- | --- | --- | --- | --- | --- |
|  |  |  |  | | | Teaching hospital sites only | |
| Author, year | Zhuo, 2018 (52) | Knights,  2012 (18) | De Briyne, 2013 (21) | Norris, 2019 (59) | Alcantara, 2021 (78) | Jacob, 2015 (27) | Ekakoro, 2019 (54) |
| Country | Australia | UK | EU countries | Australia | Portugal | UK | US |
| n | 403 | 1,121 | 1,766 | 320 | 417 | 70 | 62 |
| Percentage of companion animal veterinarians | 66% | 100% | 100% | 100% | 100% | 100% | 60% |
| Scale (very unimportant to very important) | 1 to 4 | 1 to 5 | 1 to 5 | 1 to 4 | 1 to 5 | 1 to 5 | 1 to 5 |
| Summary measure | Median | Median | Mean | Median | Median | Median | Mode |
| **Animal factors** | | | | | | | |
| Clinical signs/ symptoms | 4 | - | - | 4 | - | 5 | 4 |
| Animal's clinical history | 4 | - | - | - | - | - | - |
| Animal's condition/ immune status | 4 | - | - | - | - | - | - |
| History of antimicrobial use | 3 | - | - | 3 | - | 5 | 4 |
| Concerns about animal welfare | 3 | - | - | - | - | - | 4 |
| Patient safety | - | - | - | - | - | - | - |
| Immediate animal relief | 3 | - | - | - | - | - | - |
| Wound location | - | 3 | - | - | - | - | - |
| **Antibiotic properties** | | | | | | | |
| Likely efficacy | - | 5 | - | - | 5 | - | - |
| Spectrum of activity | - | 5 | - | 4 | - | - | - |
| Duration of activity | - | 4 | - | 3 | - | - | - |
| Potential for side effects/ adverse events | 3 | 4 | - | 3 | - | 5 | 4 |
| Bactericidal vs bacteriostatic | - | 4 | - | - | - | - | - |

In order to facilitate comparison, the scores of Jacob (2015) were inverted so that 1 = very important became 1 = very unimportant; the range of scores used by De Briyne (2013) were adjusted from 0-4 to 1-5. For example, 0 = very unimportant became 1 = very important.

**Supplementary table 4.** Studies ranking factors influencing antibiotic use (cont.)

| **Study details** | Whether or not to use antibiotics | Antibiotic choice in perioperative situations | Choice of antibiotics in general | | | | |
| --- | --- | --- | --- | --- | --- | --- | --- |
|  |  |  |  | | | Teaching hospital sites only | |
| Author, year | Zhuo, 2018 (52) | Knights,  2012 (18) | De Briyne, 2013 (21) | Norris, 2019 (59) | Alcantara, 2021 (78) | Jacob, 2015 (27) | Ekakoro, 2019 (54) |
| Country | Australia | UK | EU countries | Australia | Portugal | UK | US |
| n | 403 | 1,121 | 1,766 | 320 | 417 | 70 | 62 |
| Percentage of companion animal veterinarians | 66% | 100% | 100% | 100% | 100% | 100% | 60% |
| Scale (very unimportant to very important) | 1 to 4 | 1 to 5 | 1 to 5 | 1 to 4 | 1 to 5 | 1 to 5 | 1 to 5 |
| Summary measure | Median | Median | Mean | Median | Median | Median | Mode |
| **Other product features** | | | | | | | |
| Availability | - | - | 3.3 | 3 | 3 | 5 | 4 |
| Frequency of administration | - | - | - | 3 | - | 4 | 3 |
| Available routes of administration | - | 3 | - | 3 | - | 4 | 4 |
| Ease of administration | - | - | 3.8 | 3 | 4 | - | - |
| Medication size/ volume | - | - | - | 3 | - | 4 | 3 |
| Veterinary product license | - | 4 | - | - | 3 | - | - |
| Availability of information on drug's action | - | 3 | - | - | - | - | - |
| Shelf-life | - | 2 | - | - | - | - | - |
| Cost | - | 2 | 2.9 | 3 | 3 | 4 | 3 |
| Profit margin | - | - | 1.7 | - | - | - | - |
| Marketing offers | - | - | 1.8 | - | - | - | - |
| **Views about AMR** | | | | | | | |
| Concern about AMR | - | - | 4 | 3 | - | - | - |
| Risk of AMR in the patient | 4 | - | - | - | - | - | - |
| Concerns about AMR in animals | - | - | - | - | - | - | 4 |
| Concerns about AMR in humans | - | - | - | - | - | - | 3 |
| Community risk of AMR | 3 | - | - | - | - | - | - |
| Environmental risk of AMR | - | 3 | - | - | - | - | - |

AMR: Antimicrobial resistance. In order to facilitate comparison, the scores of Jacob (2015) were inverted so that 1 = very important became 1 = very unimportant; the range of scores used by De Briyne (2013) were adjusted from 0-4 to 1-5. For example, 0 = very unimportant became 1 = very important.

**Supplementary table 4.** Studies ranking factors influencing antibiotic use (cont.)

| **Study details** | **Whether or not to use antibiotics** | **Antibiotic choice in perioperative situations** | **Choice of antibiotics in general** | | | | |
| --- | --- | --- | --- | --- | --- | --- | --- |
|  |  |  |  | | | **Teaching hospital sites only** | |
| Author, year | Zhuo, 2018 (52) | Knights,  2012 (18) | De Briyne, 2013 (21) | Norris, 2019 (59) | Alcantara, 2021 (78) | Jacob, 2015 (27) | Ekakoro, 2019 (54) |
| Country | Australia | UK | EU countries | Australia | Portugal | UK | US |
| n | 403 | 1,121 | 1,766 | 320 | 417 | 70 | 62 |
| Percentage of companion animal veterinarians | 66% | 100% | 100% | 100% | 100% | 100% | 60% |
| Scale (very unimportant to very important) | 1 to 4 | 1 to 5 | 1 to 5 | 1 to 4 | 1 to 5 | 1 to 5 | 1 to 5 |
| Summary measure | Median | Median | Mean | Median | Median | Median | Mode |
| **Diagnostic information** | | | | | | | |
| Results of culture and sensitivity testing | 4 | - | 4.2 | 4 | - | 5 | 5 |
| Results of cytologic evaluation | - | - | 3.5 | - | - | 4 | 4 |
| **Information sources** | | | | | | | |
| Veterinarian's personal experience | 4 | - | - | 4 | - | - | - |
| SPC | - | - | 3.8 | - | - | - | - |
| SPC responsible use warnings | - | - | 3.6 | - | - | - | - |
| Guidelines /recommendations | 3 | - | 3.5 | 3 | - | - | 4 |
| Clinic policy | - | 3 | 3.4 | - | - | - | 2.5 |
| Advertisements/ promotional materials | - | - | 1.9 | 2 | - | - | 3 |
| Peer recommendations | - | - | - | - | - | 3 | - |
| **Clinic factors** | | | | | | | |
| Peer/ colleague expectations | 2 | - | - | 2 | - | - | - |
| Culture | - | - | 1.8 | - | - | - | - |
| **Owner factors** | | | | | | | |
| Owner expectations | 2 | - | 2 | 2 | - | 3 | 1 |
| Owner compliance | - | - | - | 3 | - | - | 4 |
| Fear of litigation by owner | - | - | - | - | - | - | 2 |

SPC: Summary of product characteristics. In order to facilitate comparison, the scores of Jacob (2015) were inverted so that 1 = very important became 1 = very unimportant; the range of scores used by De Briyne (2013) were adjusted from 0-4 to 1-5. For example, 0 = very unimportant became 1 = very important.

**Supplementary table 5.** Studies ranking barriers to ‘appropriate’ antibiotic use (n=2)

| **Factors explored** | **Zhuo,**  **2018 (52)** | **Hopman,**  **2019 (55)** |
| --- | --- | --- |
| Country | Australia | Netherlands |
| n | 403 | 353 |
| Percentage of companion animal veterinarians | 66% | 100% |
| Scale (very unimportant to very important) | 1 = no influence;  4 = strong influence | 1 = Completely disagree  6 = Completely agree |
| Summary measure | Median | Mode |
| **Financial/ business pressures** | | |
| My antimicrobial choices have nothing to do with higher financial profits |  | 6 |
| My antimicrobial choices have nothing to do with acquiring more clients |  | 6 |
| I freely prescribe antimicrobials because neighbouring clinics do |  | 2 |
| Fear of losing owners to different clinics if antimicrobials not prescribed | 1 |  |
| Cost of some antibiotics | 4 |  |
| **Owner interactions** | | |
| My antimicrobial choices have nothing to do with my perception of what the owner wants |  | 5 |
| My antimicrobial choices have nothing to do with what the owner wants |  | 5 |
| Pressure from owners to prescribe antimicrobials | 2 |  |
| Fear of being blamed if antibiotics are later needed | 2 |  |
| Lack of owner understanding about antimicrobials | 2 |  |
| Language/ cultural barriers when communicating with owners | 1 |  |
| I regularly encounter owner pressure to try antimicrobials before performing diagnostic tests |  | 5 |
| **Clinic environment** | | |
| My colleagues and I support each other to show restraint when prescribing antimicrobials |  | 5 |
| Our clinic policy is committed to show restraint in prescribing antimicrobials |  | 5 |
| I quite often experience pressure from colleagues and superiors to prescribe specific types of antimicrobials |  | 1 |
| Pressure from colleagues/ peers/ supervisors | 1 |  |
| Time pressures | 2 |  |
| **Diagnosis and diagnostic testing** | | |
| Difficulty of making an accurate diagnosis | 2 |  |
| Fear of missing an infection | 2 |  |
| In my clinic, we have sufficient possibilities to send samples for antimicrobial culture and susceptibility testing |  | 5 |
| I regularly encounter owners urging to try antimicrobials before performing diagnostic tests |  | 5 |
| Cost of antimicrobial culture and susceptibility testing | 3 |  |
| Lack of rapid diagnostic testing | 3 |  |
| **Information** | | |
| Lack of clear guidelines for some conditions | 2 |  |
| Lack of time to search for information | 2 |  |
| Lack of my own understanding | 1 |  |

**References**

1. Odensvik K, Grave K, Greko C. Antibacterial drugs prescribed for dogs and cats in Sweden and Norway 1990-1998. Acta Vet Scand (2001) 42:189-98. doi: 10.1186/1751-0147-42-189

2. Watson AD, Maddison JE. Systemic antibacterial drug use in dogs in Australia. Aust Vet J (2001) 79:740-6. doi: 10.1111/j.1751-0813.2001.tb10888.x

3. Rantala M, Holso K, Lillas A, Huovinen P, Kaartinen L. Survey of condition-based prescribing of antimicrobial drugs for dogs at a veterinary teaching hospital. Vet Rec (2004) 155:259-62. doi: 10.1136/vr.155.9.259

4. Heuer OE, Jensen VF, Hammerum AM. Antimicrobial drug consumption in companion animals. Emerg Infect Dis (2005) 11:344-5. doi: 10.3201/eid1102.040827

5. Holso K, Rantala M, Lillas A, Eerikainen S, Huovinen P, Kaartinen L. Prescribing antimicrobial agents for dogs and cats via university pharmacies in Finland - patterns and quality of information. Acta Vet Scand (2005) 46:87-93. doi: 10.1186/1751-0147-46-87

6. Hill PB, Lo A, Eden CA, Huntley S, Morey V, Ramsey S, et al. Survey of the prevalence, diagnosis and treatment of dermatological conditions in small animals in general practice. Vet Rec (2006) 158:533-9. doi: 10.1136/vr.158.16.533

7. Weese JS. Investigation of antimicrobial use and the impact of antimicrobial use guidelines in a small animal veterinary teaching hospital: 1995-2004. J Am Vet Med Assoc (2006) 228:553-8. doi: 10.2460/javma.228.4.553

8. Black DM, Rankin SC, King LG. Antimicrobial therapy and aerobic bacteriologic culture patterns in canine intensive care unit patients: 74 dogs (January-June 2006). J Vet Emerg Crit Care (San Antonio) (2009) 19:489-95. doi: 10.1111/j.1476-4431.2009.00463.x

9. Regula G, Torriani K, Gassner B, Stucki F, Muntener CR. Prescription patterns of antimicrobials in veterinary practices in Switzerland. J Antimicrob Chemother (2009) 63:805-11. doi: 10.1093/jac/dkp009

10. Thomson KH, Rantala MH, Viita-Aho TK, Vainio OM, Kaartinen LA. Condition-based use of antimicrobials in cats in Finland: results from two surveys. J Feline Med Surg (2009) 11:462-6. doi: 10.1016/j.jfms.2008.10.005

11. German AJ, Halladay LJ, Noble PJ. First-choice therapy for dogs presenting with diarrhoea in clinical practice. Vet Rec (2010) 167:810-4. doi: 10.1136/vr.c4090

12. Escher M, Vanni M, Intorre L, Caprioli A, Tognetti R, Scavia G. Use of antimicrobials in companion animal practice: a retrospective study in a veterinary teaching hospital in Italy. J Antimicrob Chemother (2011) 66:920-7. doi: 10.1093/jac/dkq543

13. Mateus A, Brodbelt DC, Barber N, Stark KD. Antimicrobial usage in dogs and cats in first opinion veterinary practices in the UK. J Small Anim Pract (2011) 52:515-21. doi: 10.1111/j.1748-5827.2011.01098.x

14. Radford AD, Noble PJ, Coyne KP, Gaskell RM, Jones PH, Bryan JG, et al. Antibacterial prescribing patterns in small animal veterinary practice identified via SAVSNET: the small animal veterinary surveillance network. Vet Rec (2011) 169:310. doi: 10.1136/vr.d5062

15. Wayne A, McCarthy R, Lindenmayer J. Therapeutic antibiotic use patterns in dogs: observations from a veterinary teaching hospital. J Small Anim Pract (2011) 52:310-8. doi: 10.1111/j.1748-5827.2011.01072.x

16. Baker SA, Van-Balen J, Lu B, Hillier A, Hoet AE. Antimicrobial drug use in dogs prior to admission to a veterinary teaching hospital. J Am Vet Med Assoc (2012) 241:210-7. doi: 10.2460/javma.241.2.210

17. Hughes LA, Williams N, Clegg P, Callaby R, Nuttall T, Coyne K, et al. Cross-sectional survey of antimicrobial prescribing patterns in UK small animal veterinary practice. Prev Vet Med (2012) 104:309-16. doi: 10.1016/j.prevetmed.2011.12.003

18. Knights CB, Mateus A, Baines SJ. Current British veterinary attitudes to the use of perioperative antimicrobials in small animal surgery. Vet Rec (2012) 170:646. doi: 10.1136/vr.100292

19. Murphy CP, Reid-Smith RJ, Boerlin P, Weese JS, Prescott JF, Janecko N, et al. Out-patient antimicrobial drug use in dogs and cats for new disease events from community companion animal practices in Ontario. Can Vet J (2012) 53:291-8.

20. Pleydell EJ, Souphavanh K, Hill KE, French NP, Prattley DJ. Descriptive epidemiological study of the use of antimicrobial drugs by companion animal veterinarians in New Zealand. N Z Vet J (2012) 60:115-22. doi: 10.1080/00480169.2011.643733

21. De Briyne N, Atkinson J, Pokludova L, Borriello SP, Price S. Factors influencing antibiotic prescribing habits and use of sensitivity testing amongst veterinarians in Europe. Vet Rec (2013) 173:475. doi: 10.1136/vr.101454

22. Kvaale MK, Grave K, Kristoffersen AB, Norstrom M. The prescription rate of antibacterial agents in dogs in Norway - geographical patterns and trends during the period 2004-2008. J Vet Pharmacol Ther (2013) 36:285-91. doi: 10.1111/j.1365-2885.2012.01425.x

23. De Briyne N, Atkinson J, Pokludova L, Borriello SP. Antibiotics used most commonly to treat animals in Europe. Vet Rec (2014) 175:325. doi: 10.1136/vr.102462

24. Mateus AL, Brodbelt DC, Barber N, Stark KD. Qualitative study of factors associated with antimicrobial usage in seven small animal veterinary practices in the UK. Prev Vet Med (2014) 117:68-78. doi: 10.1016/j.prevetmed.2014.05.007

25. Summers JF, Hendricks A, Brodbelt DC. Prescribing practices of primary-care veterinary practitioners in dogs diagnosed with bacterial pyoderma. BMC Vet Res (2014) 10:240. doi: 10.1186/s12917-014-0240-5

26. AVMA. Understanding companion animal practitioners' attitudes toward antimicrobial stewardship. J Am Vet Med Assoc (2015) 247:883-4. doi: 10.2460/javma.247.8.883

27. Jacob ME, Hoppin JA, Steers N, Davis JL, Davidson G, Hansen B, et al. Opinions of clinical veterinarians at a US veterinary teaching hospital regarding antimicrobial use and antimicrobial-resistant infections. J Am Vet Med Assoc (2015) 247:938-44. doi: 10.2460/javma.247.8.938

28. Buckland EL, O'Neill D, Summers J, Mateus A, Church D, Redmond L, et al. Characterisation of antimicrobial usage in cats and dogs attending UK primary care companion animal veterinary practices. Vet Rec (2016) 179:489. doi: 10.1136/vr.103830

29. Fowler H, Davis MA, Perkins A, Trufan S, Joy C, Buswell M, et al. A survey of veterinary antimicrobial prescribing practices, Washington State 2015. Vet Rec (2016) 179:651. doi: 10.1136/vr.103916

30. Lloyd D, Black C, Clark SM, Moss J, Loeffler A, Mateus A. Antimicrobial use and implementation of guidelines in UK small animal practice [poster] (2016). http://www.vetedit.com/clientFiles/resources/OneHealthBMFposterUpdated_20218_131074871673232422.pdf [accessed June 2, 2021].

31. Barbarossa A, Rambaldi J, Miraglia V, Giunti M, Diegoli G, Zaghini A. Survey on antimicrobial prescribing patterns in small animal veterinary practice in Emilia Romagna, Italy. Vet Rec (2017) 181:69. doi: 10.1136/vr.104128

32. Barzelai ID, Whittem T. Survey of systemic antimicrobial prescribing for dogs by Victorian veterinarians. Aust Vet J (2017) 95:375-85. doi: 10.1111/avj.12637

33. Burke S, Black V, Sanchez-Vizcaino F, Radford A, Hibbert A, Tasker S. Use of cefovecin in a UK population of cats attending first-opinion practices as recorded in electronic health records. J Feline Med Surg (2017) 19:687-92. doi: 10.1177/1098612X16656706

34. Chipangura JK, Eagar H, Kgoete M, Abernethy D, Naidoo V. An investigation of antimicrobial usage patterns by small animal veterinarians in South Africa. Prev Vet Med (2017) 136:29-38. doi: 10.1016/j.prevetmed.2016.11.017

35. Hardefeldt LY, Browning GF, Thursky K, Gilkerson JR, Billman-Jacobe H, Stevenson MA, et al. Antimicrobials used for surgical prophylaxis by companion animal veterinarians in Australia. Vet Microbiol (2017) 203:301-7. doi: 10.1016/j.vetmic.2017.03.027

36. Hardefeldt LY, Holloway S, Trott DJ, Shipstone M, Barrs VR, Malik R, et al. Antimicrobial Prescribing in Dogs and Cats in Australia: Results of the Australasian Infectious Disease Advisory Panel Survey. J Vet Intern Med (2017) 31:1100-7. doi: 10.1111/jvim.14733

37. Jessen LR, Sorensen TM, Lilja ZL, Kristensen M, Hald T, Damborg P. Cross-sectional survey on the use and impact of the Danish national antibiotic use guidelines for companion animal practice. Acta Vet Scand (2017) 59:81. doi: 10.1186/s13028-017-0350-8

38. Sarrazin S, Vandael F, Van Cleven A, De Graef E, de Rooster H, Dewulf J. The impact of antimicrobial use guidelines on prescription habits in fourteen Flemish small animal practices. De impact van advies omtrent het gebruik van antimicrobiële middelen op het voorschrijfgedrag in veertien Vlaamse praktijken voor kleine huisdieren. *Vlaams Diergeneeskundig Tijdschrift* (2017) 86:173-82.

39. Singleton DA, Sanchez-Vizcaino F, Dawson S, Jones PH, Noble PJM, Pinchbeck GL, et al. Patterns of antimicrobial agent prescription in a sentinel population of canine and feline veterinary practices in the United Kingdom. Vet J (2017) 224:18-24. doi: 10.1016/j.tvjl.2017.03.010

40. Cartelet C, Hobson-West P, Raman S, Millar K. Antimicrobial resistance and companion animal medicine: examining constructions of responsibility In: Springer S, Grimm H, editors. 14th Congress of the European Society for Agricultural and Food Ethics; 2018 Jun 13-16; Vienna, Austria. The Netherlands: Wageningen Academic Publishers (2018). p. 296–301.

41. Currie K, King C, Nuttall T, Smith M, Flowers P. Expert consensus regarding drivers of antimicrobial stewardship in companion animal veterinary practice: a Delphi study. Vet Rec (2018) 182:691. doi: 10.1136/vr.104639

42. Dyar OJ, Hills H, Seitz LT, Perry A, Ashiru-Oredope D. Assessing the Knowledge, Attitudes and Behaviors of Human and Animal Health Students towards Antibiotic Use and Resistance: A Pilot Cross-Sectional Study in the UK. Antibiotics (Basel) (2018) 7:10. doi: 10.3390/antibiotics7010010

43. Gomez-Poveda B, Moreno MA. Antimicrobial Prescriptions for Dogs in the Capital of Spain. Front Vet Sci (2018) 5:309. doi: 10.3389/fvets.2018.00309

44. Hardefeldt L, Nielsen T, Crabb H, Gilkerson J, Squires R, Heller J, et al. Veterinary Students' Knowledge and Perceptions About Antimicrobial Stewardship and Biosecurity-A National Survey. Antibiotics (Basel) (2018) 7:34. doi: 10.3390/antibiotics7020034

45. Hardefeldt LY, Gilkerson JR, Billman-Jacobe H, Stevenson MA, Thursky K, Bailey KE, et al. Barriers to and enablers of implementing antimicrobial stewardship programs in veterinary practices. J Vet Intern Med (2018) 32:1092-9. doi: 10.1111/jvim.15083

46. Hardefeldt LY, Selinger J, Stevenson MA, Gilkerson JR, Crabb H, Billman-Jacobe H, et al. Population wide assessment of antimicrobial use in dogs and cats using a novel data source - A cohort study using pet insurance data. Vet Microbiol (2018) 225:34-9. doi: 10.1016/j.vetmic.2018.09.010

47. Hopman NEM, Hulscher M, Graveland H, Speksnijder DC, Wagenaar JA, Broens EM. Factors influencing antimicrobial prescribing by Dutch companion animal veterinarians: A qualitative study. Prev Vet Med (2018) 158:106-13. doi: 10.1016/j.prevetmed.2018.07.013

48. King C, Smith M, Currie K, Dickson A, Smith F, Davis M, et al. Exploring the behavioural drivers of veterinary surgeon antibiotic prescribing: a qualitative study of companion animal veterinary surgeons in the UK. BMC Vet Res (2018) 14:332. doi: 10.1186/s12917-018-1646-2

49. Smith M, King C, Davis M, Dickson A, Park J, Smith F, et al. Pet owner and vet interactions: exploring the drivers of AMR. Antimicrob Resist Infect Control (2018) 7:46. doi: 10.1186/s13756-018-0341-1

50. Sorensen TM, Bjornvad CR, Cordoba G, Damborg P, Guardabassi L, Siersma V, et al. Effects of Diagnostic Work-Up on Medical Decision-Making for Canine Urinary Tract Infection: An Observational Study in Danish Small Animal Practices. J Vet Intern Med (2018) 32:743-51. doi: 10.1111/jvim.15048

51. Van Cleven A, Sarrazin S, de Rooster H, Paepe D, Van der Meeren S, Dewulf J. Antimicrobial prescribing behaviour in dogs and cats by Belgian veterinarians. Vet Rec (2018) 182:324. doi: 10.1136/vr.104316.

52. Zhuo A, Labbate M, Norris JM, Gilbert GL, Ward MP, Bajorek BV, et al. Opportunities and challenges to improving antibiotic prescribing practices through a One Health approach: results of a comparative survey of doctors, dentists and veterinarians in Australia. BMJ Open (2018) 8:e020439. doi: 10.1136/bmjopen-2017-020439

53. Dickson A, Smith M, Smith F, Park J, King C, Currie K, et al. Understanding the relationship between pet owners and their companion animals as a key context for antimicrobial resistance-related behaviours: an interpretative phenomenological analysis. Health Psychol Behav Med (2019) 7:45-61. doi: 10.1080/21642850.2019.1577738

54. Ekakoro JE, Okafor CC. Antimicrobial use practices of veterinary clinicians at a veterinary teaching hospital in the United States. Vet Anim Sci (2019) 7:100038. doi: 10.1016/j.vas.2018.09.002

55. Hopman NEM, Mughini-Gras L, Speksnijder DC, Wagenaar JA, van Geijlswijk IM, Broens EM. Attitudes and perceptions of Dutch companion animal veterinarians towards antimicrobial use and antimicrobial resistance. Prev Vet Med (2019) 170:104717. doi: 10.1016/j.prevetmed.2019.104717

56. Hopman NEM, van Dijk MAM, Broens EM, Wagenaar JA, Heederik DJJ, van Geijlswijk IM. Quantifying Antimicrobial Use in Dutch Companion Animals. Front Vet Sci (2019) 6:158. doi: 10.3389/fvets.2019.00158

57. Hopman NEM, Portengen L, Hulscher M, Heederik DJJ, Verheij TJM, Wagenaar JA, et al. Implementation and evaluation of an antimicrobial stewardship programme in companion animal clinics: A stepped-wedge design intervention study. PLoS One (2019) 14:e0225124. doi: 10.1371/journal.pone.0225124

58. Hopman NEM, Portengen L, Heederik DJJ, Wagenaar JA, Van Geijlswijk IM, Broens EM. Time trends, seasonal differences and determinants of systemic antimicrobial use in companion animal clinics (2012-2015). Vet Microbiol (2019) 235:289-94. doi: 10.1016/j.vetmic.2019.07.016

59. Norris JM, Zhuo A, Govendir M, Rowbotham SJ, Labbate M, Degeling C, et al. Factors influencing the behaviour and perceptions of Australian veterinarians towards antibiotic use and antimicrobial resistance. PLoS One (2019) 14:e0223534. doi: 10.1371/journal.pone.0223534

60. Redding LE, Cole SD. Pet owners' knowledge of and attitudes toward the judicious use of antimicrobials for companion animals. J Am Vet Med Assoc (2019) 254:626-35. doi: 10.2460/javma.254.5.626

61. Redding LE, Cole SD. Posters Have Limited Utility in Conveying a Message of Antimicrobial Stewardship to Pet Owners. Front Vet Sci (2019) 6:421. doi: 10.3389/fvets.2019.00421

62. Schmitt K, Lehner C, Schuller S, Schupbach-Regula G, Mevissen M, Peter R, et al. Antimicrobial use for selected diseases in cats in Switzerland. BMC Vet Res (2019) 15:94. doi: 10.1186/s12917-019-1821-0

63. Singleton DA, Arsevska E, Smyth S, Barker EN, Jewell C, Brant B, et al. Small animal disease surveillance: gastrointestinal disease, antibacterial prescription and Tritrichomonas foetus. Vet Rec (2019) 184:211-6. doi: 10.1136/vr.l722

64. Singleton DA, McGarry J, Torres JR, Killick D, Jewell C, Smyth S, et al. Small animal disease surveillance 2019: pruritus, pharmacosurveillance, skin tumours and flea infestations. Vet Rec (2019) 185:470-5. doi: 10.1136/vr.l6074.

65. Singleton DA, Noble PJM, Sanchez-Vizcaino F, Dawson S, Pinchbeck GL, Williams NJ, et al. Pharmaceutical Prescription in Canine Acute Diarrhoea: A Longitudinal Electronic Health Record Analysis of First Opinion Veterinary Practices. Front Vet Sci (2019) 6:218. doi: 10.3389/fvets.2019.00218.

66. Singleton DA, Stavisky J, Jewell C, Smyth S, Brant B, Sanchez-Vizcaino F, et al. Small animal disease surveillance 2019: respiratory disease, antibiotic prescription and canine infectious respiratory disease complex. Vet Rec (2019) 184:640-5. doi: 10.1136/vr.l3128

67. Hardefeldt L, Hur B, Verspoor K, Baldwin T, Bailey KE, Scarborough R, et al. Use of cefovecin in dogs and cats attending first-opinion veterinary practices in Australia. Vet Rec (2020) 187:e95. doi: 10.1136/vr.105997

68. Hubbuch A, Schmitt K, Lehner C, Hartnack S, Schuller S, Schupbach-Regula G, et al. Antimicrobial prescriptions in cats in Switzerland before and after the introduction of an online antimicrobial stewardship tool. BMC Vet Res (2020) 16:229 doi: 10.1186/s12917-020-02447-8

69. Hur BA, Hardefeldt LY, Verspoor KM, Baldwin T, Gilkerson JR. Describing the antimicrobial usage patterns of companion animal veterinary practices; free text analysis of more than 4.4 million consultation records. PLoS One (2020) 15:e0230049. doi: 10.1371/journal.pone.0230049

70. Joosten P, Ceccarelli D, Odent E, Sarrazin S, Graveland H, Van Gompel L, et al. Antimicrobial Usage and Resistance in Companion Animals: A Cross-Sectional Study in Three European Countries. Antibiotics (Basel) (2020) 9:87. doi: 10.3390/antibiotics9020087

71. Lehner C, Hubbuch A, Schmitt K, Schuepbach-Regula G, Willi B, Mevissen M, et al. Effect of antimicrobial stewardship on antimicrobial prescriptions for selected diseases of dogs in Switzerland. J Vet Intern Med (2020) 34:2418-31. doi: 10.1111/jvim.15906

72. Lutz B, Lehner C, Schmitt K, Willi B, Schupbach G, Mevissen M, et al. Antimicrobial prescriptions and adherence to prudent use guidelines for selected canine diseases in Switzerland in 2016. Vet Rec Open (2020) 7:e000370. doi: 10.1136/vetreco-2019-000370

73. Robbins SN, Goggs R, Lhermie G, Lalonde-Paul DF, Menard J. Antimicrobial Prescribing Practices in Small Animal Emergency and Critical Care. Front Vet Sci (2020) 7:110. doi: 10.3389/fvets.2020.00110

74. Singleton DA, Pinchbeck GL, Radford AD, Arsevska E, Dawson S, Jones PH, et al. Factors Associated with Prescription of Antimicrobial Drugs for Dogs and Cats, United Kingdom, 2014-2016. Emerg Infect Dis (2020) 26:1778-91. doi: 10.3201/eid2608.191786

75. Stallwood J, Shirlow A, Hibbert A. A UK-based survey of cat owners' perceptions and experiences of antibiotic usage. J Feline Med Surg (2020) 22:69-76 doi: 10.1177/1098612X19826353

76. Tompson AC, Chandler CIR, Mateus ALP, O'Neill DG, Chang Y-M, Brodbelt DC. What drives antimicrobial prescribing for companion animals? A mixed-methods study of UK veterinary clinics. Prev Vet Med (2020) 183:105117. doi: 10.1016/j.prevetmed.2020.105117

77. Valiakos G, Pavlidou E, Zafeiridis C, Tsokana CN, Del Rio Vilas VJ. Antimicrobial practices among small animal veterinarians in Greece: a survey. One Health Outlook (2020) 2:7. doi: 10.1186/s42522-020-00013-8

78. Alcantara GLC, Pinello KC, Severo M, Niza-Ribeiro J. Antimicrobial resistance in companion animals - Veterinarians' attitudes and prescription drivers in Portugal. Comp Immunol Microbiol Infect Dis (2021) 76:101640. doi: 10.1016/j.cimid.2021.101640

79. Chirollo C, Nocera FP, Piantedosi D, Fatone G, Della Valle G, De Martino L, et al. Data on before and after the Traceability System of Veterinary Antimicrobial Prescriptions in Small Animals at the University Veterinary Teaching Hospital of Naples. Animals (Basel) (2021) 11:913. doi: 10.3390/ani11030913

80. Galarce N, Arriagada G, Sanchez F, Venegas V, Cornejo J, Lapierre L. Antimicrobial Use in Companion Animals: Assessing Veterinarians' Prescription Patterns through the First National Survey in Chile. Animals (Basel) (2021) 11:348. doi: 10.3390/ani11020348

81. Lavigne SH, Louis S, Rankin SC, Zaoutis TE, Szymczak JE. How companion animal veterinarians in the United States perceive financial constraints on antibiotic decision-making. Vet Rec (2021) 188:e62. doi: 10.1002/vetr.62.

82. Samuels R, Qekwana DN, Oguttu JW, Odoi A. Antibiotic prescription practices and attitudes towards the use of antimicrobials among veterinarians in the City of Tshwane, South Africa. PeerJ (2021) 9:e10144-e. doi: 10.7717/peerj.10144

83. Singleton DA, Rayner A, Brant B, Smyth S, Noble PM, Radford AD, et al. A randomised controlled trial to reduce highest priority critically important antimicrobial prescription in companion animals. Nat Commun (2021) 12:1593. doi: 10.1038/s41467-021-21864-3.
